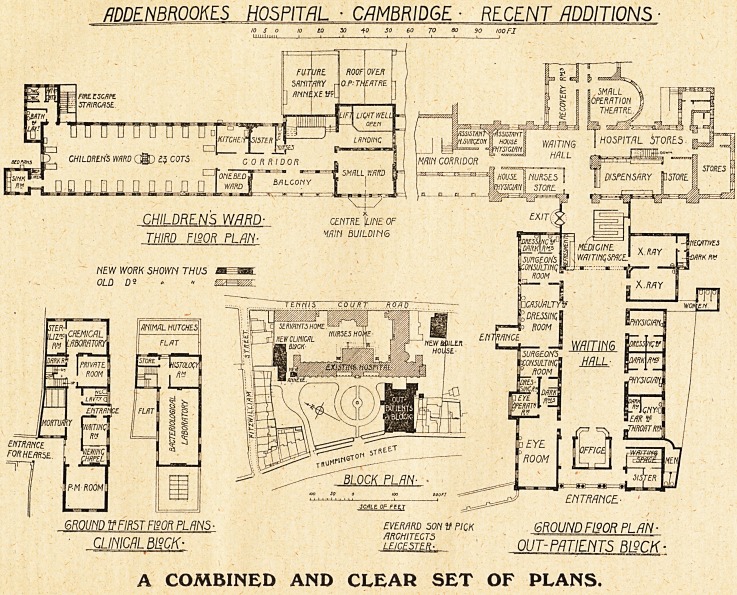# Addenbrooke's Hospital, Cambridge

**Published:** 1917-02-24

**Authors:** 


### HOSPITAL ARCHITECTURE AND CONSTRUCTION.

### Addenbrooke's Hospital, Cambridge.

The works recently carried out under the direction of Messrs. Everard, Son and Pick, architects, of Leicester, form part of a larger scheme, the completion of which has had to be postponed. They comprise a new out-patient department, a new sanitary annex, and the remodelling of the existing sanitary annex to the north ward wing, new wards on top of the existing building, a new clinical block, and a new boiler-house and engineering plant.

The out-patient department faces Trumpington Street, and is a one-storey building. The patients enter by a recessed portico facing the street, in which are two entrance-doors, one for new the other for old patients. The office is set midway between the two entrance passages, so that old patients have their books handed out to them at one window while new patients are registered and obtain their books at the other window. From thence they pass to a long waiting-hall. A small waiting-space close to the entrance, with two rooms for "Sister" attached, seems to suggest the work of an almoner.

The various consulting-rooms are arranged on either side of the waiting-hall. On the south side, beginning at the entrance end, is a room used jointly by the gynæcologist and the surgeon to the ear and throat department. Next to this are two consulting-rooms for physicians, each with dressing and dark rooms; and at the extreme end is the $x$-ray department, consisting of two rooms and a dark-room.

On the north side, beginning at the entrance end, is the eye department, comprising a large room for refraction and general work, a small operating-room, a dark-room with three compartments, and a dressing-room. The dressing-room and dark-rooms are also accessible to the surgeons' consulting-room, which adjoins the eye department. Next to the surgeons' consulting-room is a large casualty dressing-room with a separate entrance-porch from the front drive, and adjoining this is a second consulting-room for surgeon, with dressing and dark room.

At the extreme end of the waiting-hall is a refreshment counter and a railed-off seated space for patients waiting for medicine. The dispensary, which is in the old building, was formerly the out-patients' waiting-hall, and has now been fitted up for its new purpose.

Separate sanitary annexes are arranged for male and female patients, with complete open-air disconnection.

The rooms in the old hospital formerly used for out-patient work have been converted into stores.

The old sanitary block to the north wing has been remodelled and provided with entirely new fittings; the walls have been lined with tiles and the floors reconstructed and finished with terrazzo. A new block containing sink-rooms has been built at the north-west corner. On each floor is a spacious room fitted with the necessary sinks and provided with a ventilated cupboard for bed-pans. The walls and floor are finished in a similar way to the other sanitary annex. It so often happens that we have to criticise adversely the inadequate space allotted to sink-room work that it is with special pleasure that we recognise in the present instance the fact that the architects know what the work is and provide accordingly.

On the top floor of the north wing of the existing building a new floor of wards with their offices has been constructed.

In carrying out this work the old walls were found to be of very poor construction, and it became necessary to strengthen the whole building. This was done by inserting a ferro-concrete band on the top of the old walls, which forms a box-like support for the new work, the weight being distributed evenly over all the old walls and foundations. The large children's ward contains twenty-five cots, and a small one-bed ward is placed next to the large ward. There is also another small ward, and the balcony on the floor below is repeated here. A new electrically-driven lift has been installed, running from the basement to the new top floor. Much renovation work has been done to the old hospital, of which perhaps the most useful is the construction of ducts for the various pipes, of sufficient size for men to walk through from end to end.

The new clinical block is the gift of Mrs. Bonnett, who has also generously endowed it as a memorial to her late husband, for many years secretary to the hospital. It is a detached building two storeys high, and contains, on the ground floor, *post-mortem* room, mortuary, viewing and waiting room for friends, chemical laboratory with sterilising-room, dark-room, and private room. On the upper floor is a large laboratory for bacteriology, a smaller one for histology, and an animal-house on the roof of the ground floor.

Before the recent alterations the engineering plant of the hospital was apparently in a chaotic state. Several boilers were in existence in different parts of the building, and the work, while inefficiently performed, must have been very costly. The only wise course was adopted; the whole of the old plant was scrapped and two new boilers in a new boiler-house provided, one of which does efficiently the whole work of the hospital.

## Figures and Tables

**Figure f1:**